# Supplementary material for: Prevention of Encrustation on Ureteral Stents: Which Surface Parameters Provide Guidance for the Development of Novel Stent Materials?
Source: Polymers (Basel). 2020 Mar 3;12(3):558. doi: 10.3390/polym12030558 (PMC7182952; doi:10.3390/polym12030558)
Supplement: Supplementary file 1 [file polymers-12-00558-s001.pdf]

Supplementary Materials

Prevention of encrustation on ureteral stents: Which surface parameters provide guidance for the development of novel stent materials?

By Henrike Rebl [a]*, Jürgen Renner [b], Wolfgang Kram [c], Armin Springer [d], Nele Fritsch [a], Harald Hansmann [b], Oliver W. Hakenberg [c] and J. Barbara Nebe [a]

pH curve

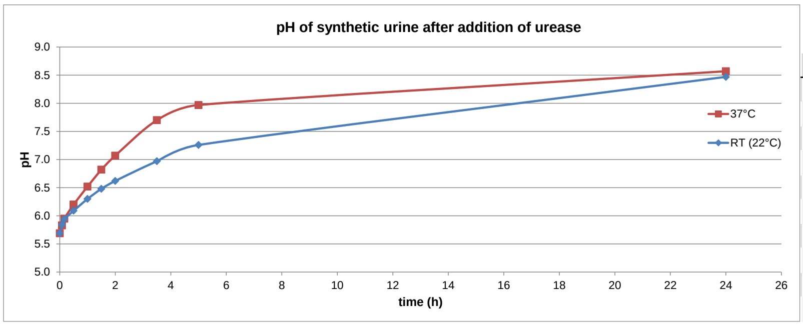

time (h)	37°C	RT (22°C)
0.00	5.7	5.7
0.08	5.8	5.8
0.17	6.0	5.9
0.50	6.2	6.1
1.00	6.5	6.3
1.50	6.8	6.5
2.00	7.1	6.6
3.50	7.7	7.0
5.00	8.0	7.3
24.00	8.6	8.5

Figure S1. pH of synthetic urine: pH measurements of the synthetic urine inside the reaction chamber of the in vitro encrustation system. The pH rises as soon as the decomposition of urea by the enzyme urease starts. By the constant exchange of the liquid a pH equilibrium is reached after a few hours.

Contact angle

		Contact angle				
		pH 5.6		pH 6.5		
		mean	SD	mean	SD	No. of measurements
1	Elastollan 1185 A	84	15	85	13	5
2	Carbothane PC 3585A	127	4	92	2	3
3	Pelletane 2363 80A	102	4	105	5	5
4	Tecoflex EG 60D	112	3	111	2	5
5	Tecophilic HP-93A-100	106	5	104	6	6
6	Tecothane TT 1095	115	3	104	4	5
7	Styroflex 2G66	99	3	85	5	4
8	Greenflex FL 65	79	5	86	1	4

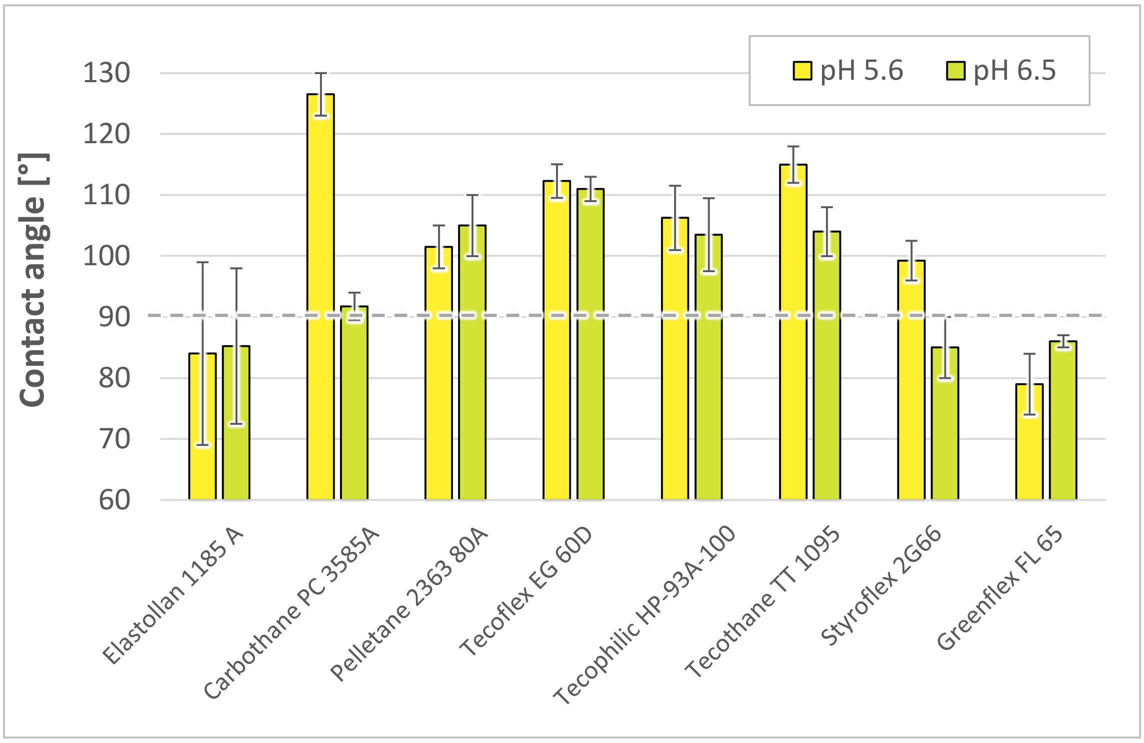

Figure S2. Contact angle: Contact angle of the various polymers measured with synthetic urine at different pH values. Mean +/- SD

EDX

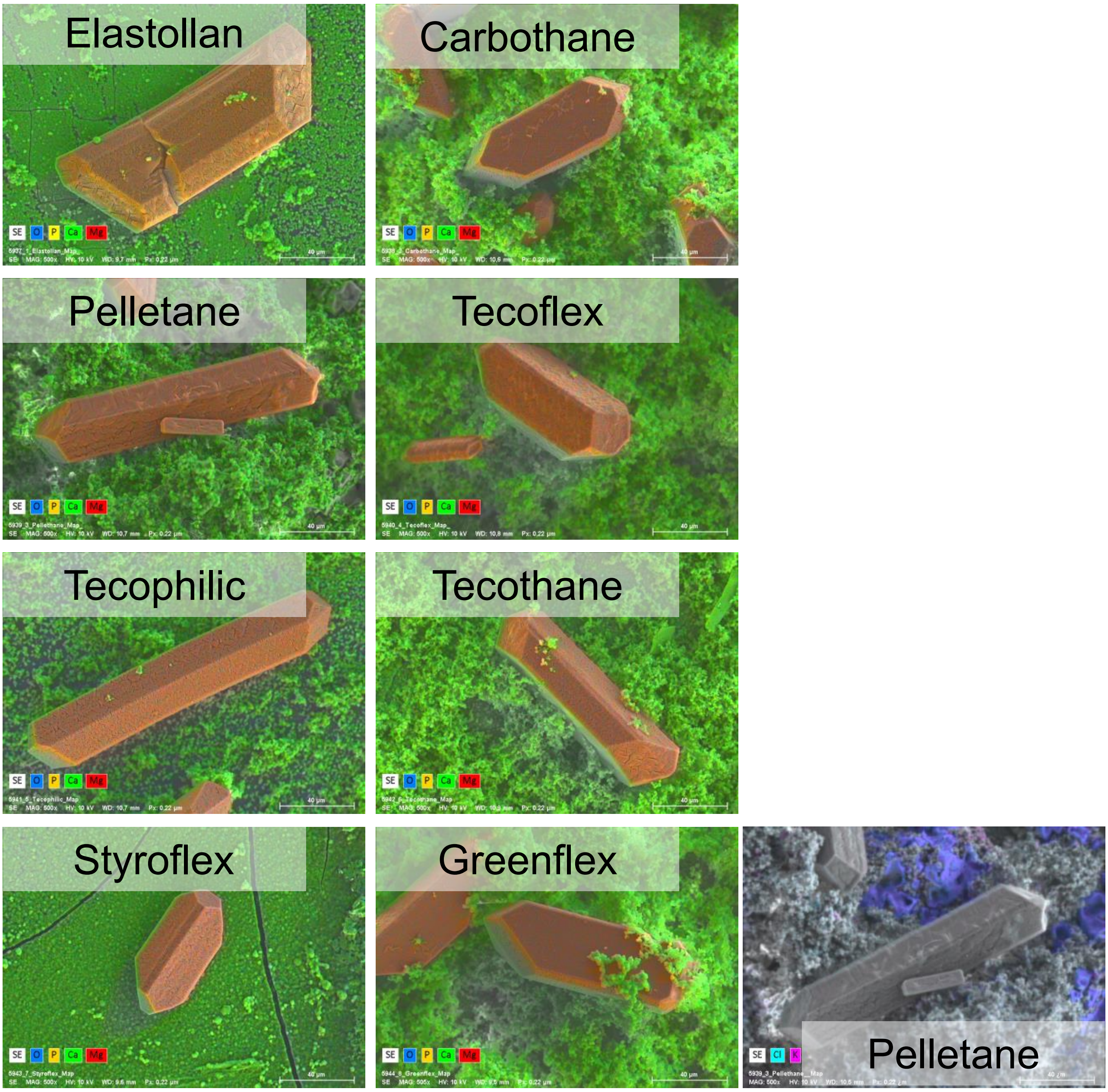

Figure S3. EDX analyses: Overview of the mineral deposits on the polymer surfaces. We aimed at showing carbonate apatite and struvite crystals on all images, even though struvite crystals are very rare on Elastollan, Styroflex and Greenflex. The green (calcium phosphate/apatite) matrix is rather thin on Elastollan, Styroflex and Greenflex – Group 1 – sparsely encrusted polymers.

On the other polymers one can clearly see a multi-layered matrix of apatite crystallites, with struvite crystals embedded in these thick depositions. Solely on Pelletane we additionally found potassium chloride crystals randomly distributed over the surface and embedded in the calcium phosphate crystallites. (FE-SEM Merlin® VP Compact (Zeiss) with EDX detector (XFlash 6/30, Bruker)

FTIR

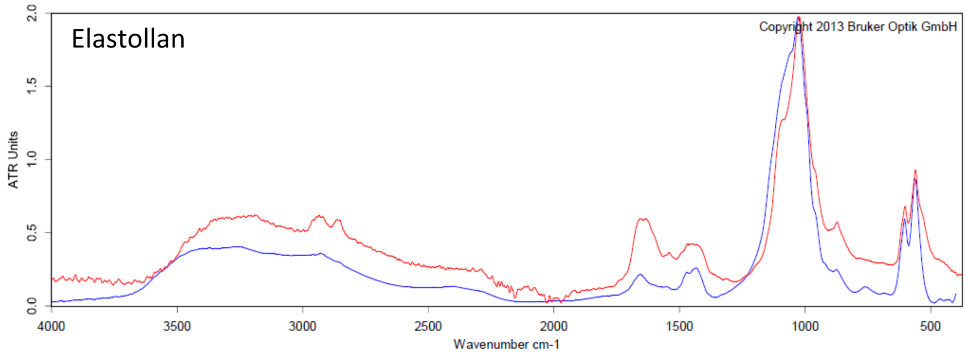

Color	Hit quality	Compound name	Compound ratio [%]
red		Elastollan	
blue	776	whitlockite<br>carbonate apatite<br>struvite	60<br>20<br>20

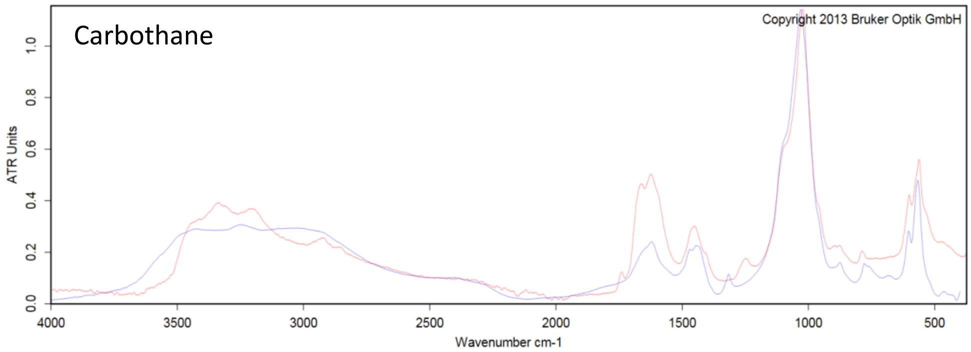

Color	Hit quality	Compound name	Compound ratio [%]
red		Carbothane	
blue	928	carbonate apatite<br>Struvite<br>whewellite	50<br>40<br>10

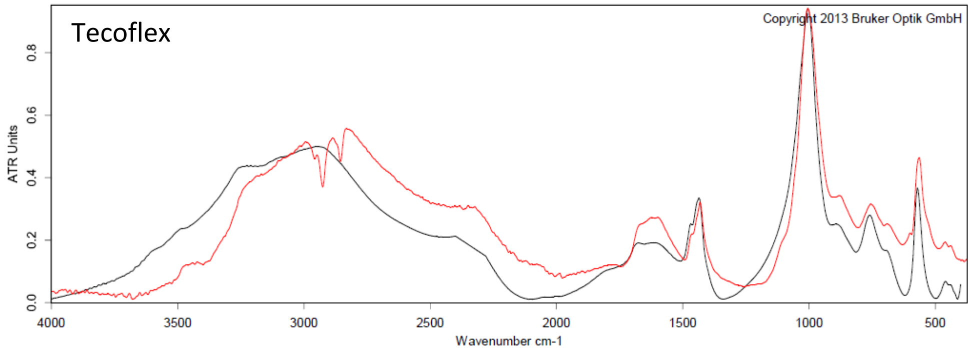

Color	Hit quality	Compound name	Compound ratio [%]
red		Tecoflex	
black	663	struvite<br>carbonate apatite	95<br>5

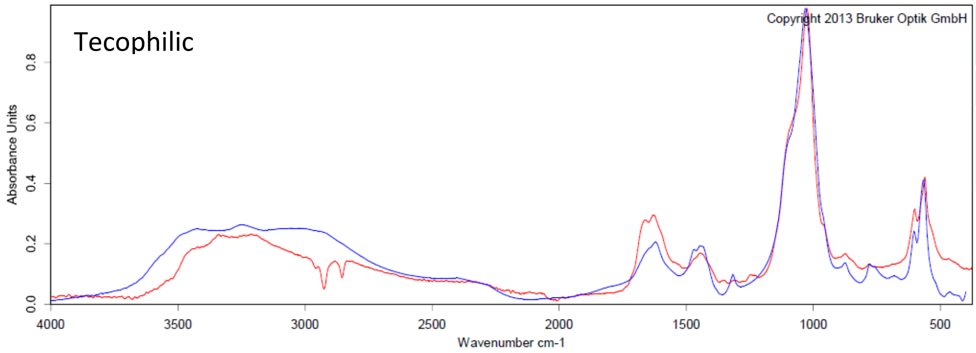

Color	Hit quality	Compound name	Compound ratio [%]
red		Tecophilic	
blue	699	carbonate apatite<br>struvite<br>whewellite	50<br>40<br>10

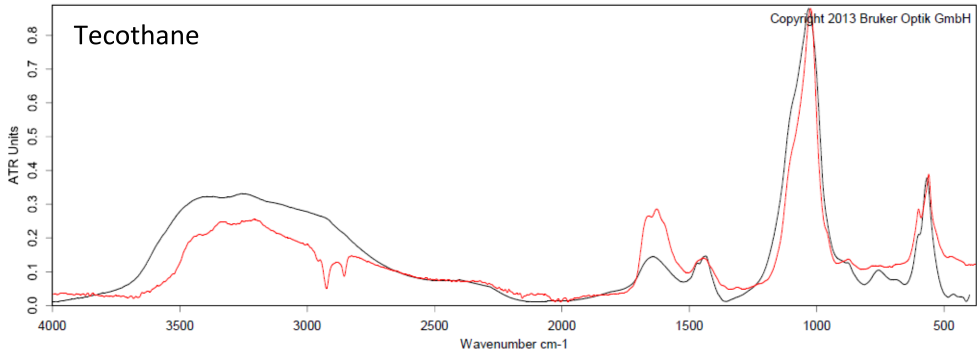

Color	Hit quality	Compound name	Compound ratio [%]
red		Tecothane	
black	757	amorph. carbonated ca-phosphate<br>struvite<br>carbonate apatite	50<br>30<br>20

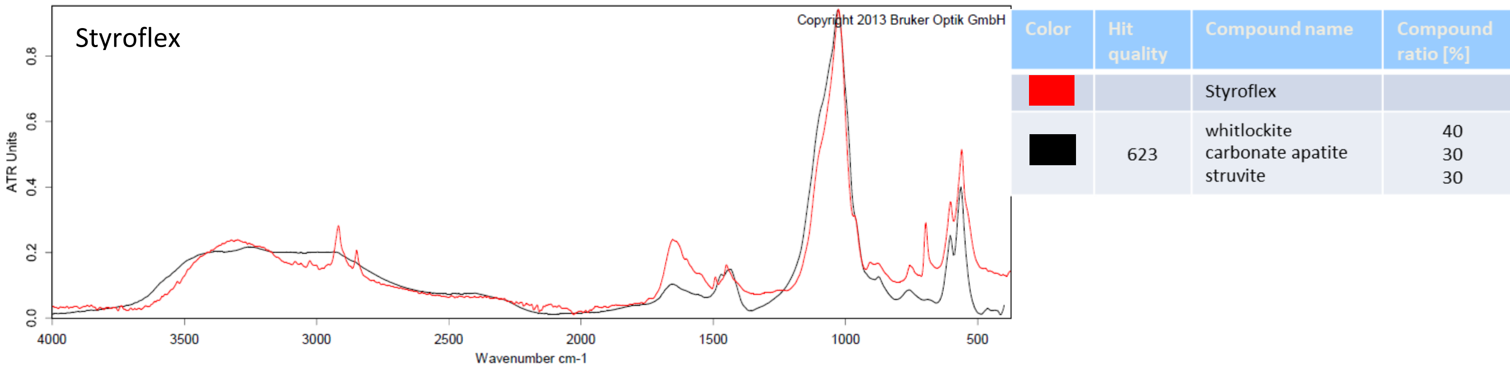

Color	Hit quality	Compound name	Compound ratio [%]
red		Styroflex	
black	623	whitlockite<br>carbonate apatite<br>struvite	40<br>30<br>30

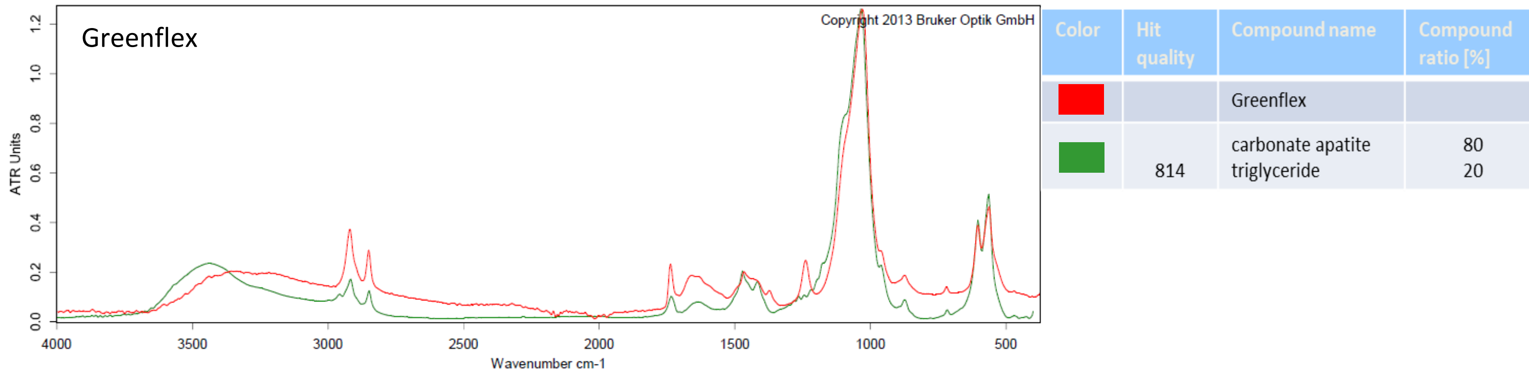

Color	Hit quality	Compound name	Compound ratio [%]
red		Greenflex	
green	814	carbonate apatite<br>triglyceride	80<br>20

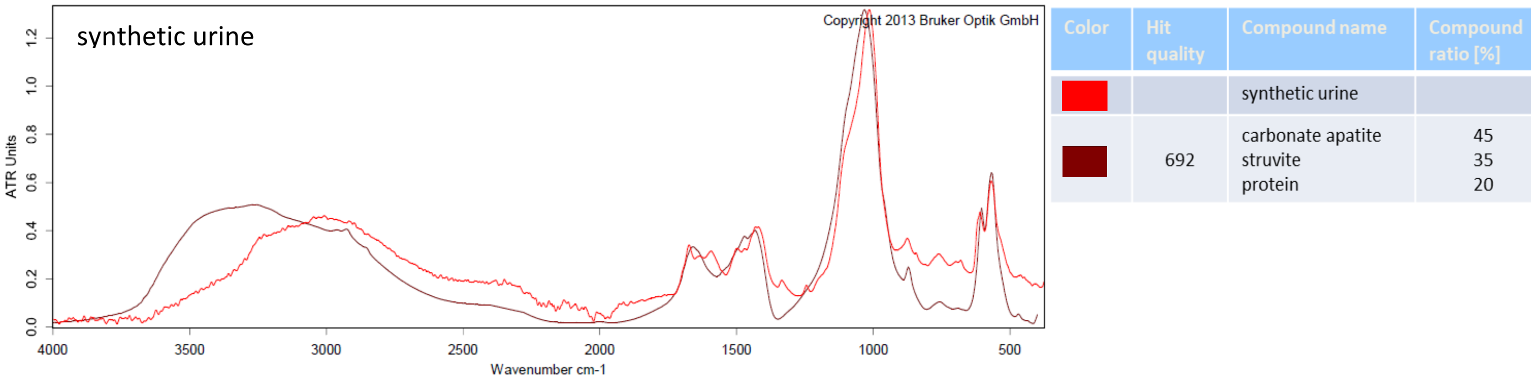

Color	Hit quality	Compound name	Compound ratio [%]
red		synthetic urine	
dark red	692	carbonate apatite<br>struvite<br>protein	45<br>35<br>20

Figure S4. FTIR measurements of the mineral deposits on the polymer samples (Elastollan, Carbothane, Pelletane, Tecoflex, Thecophilic, Tecothane, Styroflex and Greenflex). The measured curve is colored in red. The best fit of the OPUS library (Bruker) with percentual composition is indicated in the table on the right with the respective color in the graph and in the table.
